# Genome-wide analysis of key gene families in RNA silencing and their responses to biotic and drought stresses in adzuki bean

**DOI:** 10.1186/s12864-023-09274-9

**Published:** 2023-04-12

**Authors:** Yongqiang Li, Enze Ma, Kai Yang, Bo Zhao, Yisong Li, Ping Wan

**Affiliations:** 1 Key Laboratory for Northern Urban Agriculture of Ministry of Agriculture Rural Affairs, College of Biological Science and Resources Environment, Beijing University of Agriculture, HuilongguanBeinonglu 7, Changping District, Beijing, 102206 China; 2 Beijing Key Laboratory of New Technology in Agricultural Application, College of Plant Science and Technology, Beijing University of Agriculture, HuilongguanBeinonglu 7, Changping District, Beijing, 102206 China

**Keywords:** Adzuki bean (*Vigna angularis*), DCL, AGO, RDR, Gene family, Biotic and drought stresses

## Abstract

**Background:**

In plants, RNA silencing is an important conserved mechanism to regulate gene expression and combat against abiotic and biotic stresses. Dicer-like (DCL) and Argonaute (AGO) proteins and RNA-dependent RNA polymerase (RDR) are the core elements involved in gene silencing and their gene families have been explored in many plants. However, these genes and their responses to stresses have not yet been well characterized in adzuki bean.

**Results:**

A total of 11 AGO, 7 DCL and 6 RDR proteins were identified, and phylogenetic analyses of these proteins showed that they clustered into six, four and four clades respectively. The expression patterns of these genes in susceptible or resistant adzuki bean cultivars challenged with drought, bean common mosaic virus and *Podosphaera xanthii* infections were further validated by quantitative RT-PCR. The different responses of these proteins under abiotic and biotic stresses indicated their specialized regulatory mechanisms.

**Conclusions:**

In this study, 24 genes of the DCL, AGO and RDR gene families in adzuki bean were identified, and the sequence characterization, structure of the encoded proteins, evolutionary relationship with orthologues in other legumes and gene expression patterns under drought and biotic stresses were primarily explored, which enriched our understanding of these genes in adzuki bean. Our findings provide a foundation for the comparative genomic analyses of RNA silencing elements in legume plants and further new insights into the functional complexity of RNA silencing in the response to various stresses in adzuki bean.

**Supplementary Information:**

The online version contains supplementary material available at 10.1186/s12864-023-09274-9.

## Introduction

The adzuki bean (*Vigna angularis*), in the *Ceratotropis* subgenus, under the *papilionoid* subfamily of the *Fabaceae*, is an important legume crop with high easily digestible protein content and extremely low fat content [1, 2]. In China, adzuki bean has been domesticated for 12,000 years [1] and is now cultivated in over 30 countries, mainly in eastern and northern Asia [1, 3], due to its broad adaptability to poor soil and strong nitrogen fixation capability which contributions greatly to soil condition improvement [3, 4]. China is the largest producer of adzuki bean with approximately 670,000 ha [5]. In China, adzuki bean is used as a traditional healthy food that meets consumers′ health consciousness and is recommended as a suitable food for diabetic patients due to its rich content of phenolic compounds, flavonoids, vitamin A, vitamin B, iron, zinc, and folate [6, 7]. Furthermore, adzuki bean is also a traditional medicine used as a diuretic and antidote to alleviate symptoms of dropsy and beriberi [8]. Similar to other legume plants, adzuki beans are also affected by various abiotic (drought, flood and low temperature) and biotic (pests, fungi, bacteria and viruses) stresses, which may reduce yields by up to 20% [3]. Powdery mildew is a common fungal disease threatening the cultivation of adzuki bean in China especially under high humidity or high temperature [9]. Bean common mosaic virus (BCMV) is one of the most damaging viruses infecting legume plants with worldwide distribution and has been reported in adzuki bean in China with serious symptoms [10, 11]. For abiotic stress, drought is one of the most devastating environmental stresses that restricts the root and shoot growth and the final productivity [12].

Plant small noncoding RNAs (ncRNAs) play an important role in regulating morphological development, nutrient metabolism and responses to diverse stresses caused by biotic or abiotic factors [13, 14]. Among the various plant ncRNAs, microRNAs (miRNAs) and small interfering RNAs (siRNAs) are the two major classes. MiRNAs can negatively regulate endogenous gene expression through either target mRNA cleavage, translational repression, or DNA methylation, and are inextricably linked to a plethora of developmental processes, such as meristem development, establishment of lateral organ polarity and boundaries, vegetative and reproductive organ growth and leaf pattern formation as well as abiotic and biotic stress responses [14, 15]. They are generated by different Dicer-like (DCL), Argonaute (AGO), and RNA-dependent RNA polymerase (RDR) proteins. DCLs are typical members of RNase III family with endoribonuclease activity and cleave double-stranded RNAs (dsRNAs) into small RNA duplexes with 21–24 nucleotides. All the DCL proteins were conserved with the domains of DEAD box helicase (DExD), helicase conserved C-terminal domain (HelC), Dicer dimer (Dd), Piwi/Argonaute/Zwille (PAZ), RNaseIII C (RIBO III), and double-stranded RNA-binding (dsRB). As the core proteins forming the RNA induced silencing complex, AGOs are guided by small RNAs to their targets by perfect or near perfect sequence complementarity, which results in cleavage of target mRNA, initiation or elongation block to repress translation, or chromatin modification including cytosine and/or histone methylation, or heterochromatin formation [16, 17]. Specific domains including DUF1785, PAZ, MID and PIWI were also found to be conserved in plant AGO proteins [18]. The small RNA signal was amplified by plant encoded RDRs, resulting in enhanced RNAi potency. The conserved RNA-dependent RNA polymerase catalytic domain is required for initiation and amplification of the silencing signal [19].

DCL, AGO and RDR duplications are known to exist in plants. In Arabidopsis, four DCLs exist and are functionally specialized in producing different types of small RNAs with varying sizes [20]. Ten AGO proteins exist in Arabidopsis and some of them have been well studied. AGO1 is associated with most miRNAs and some siRNAs such as trans-acting siRNAs (ta-siRNAs) to regulate plant development and stress adaptations through target mRNA cleavage and/or translation inhibition [21]. AGO6 is partly redundant with AGO4 and involved in specific siRNA accumulation related to heterochromatin, DNA methylation and transcriptional gene silencing [22]. AGO7 has been demonstrated to bind miR390 and trigger the ta*-*siRNA generation [23]; in Arabidopsis it is required for the AtlsiRNA-1 accumulation and contributes to effector triggered immunity [24]. For RDRs in Arabidopsis, RDR2 converts ssRNAs generated from repetitive DNAs to precursor dsRNAs of repeat-associated siRNAs, while RDR6 produces the ta-siRNA precursors [25].

Gene families encoding the DCL, RDR and AGO proteins, the core components of RNA silencing, have been identified in many plant species including *Brassica napus* [26], *Glycine max, Sorghum bicolor* [27], *Cicer arietinum*, *Cajanus cajan*, and *Arachis hypogaea* [28]. As an important diploid pulse crop (2n = 2x = 22), adzuki bean can be used as a model species for non-oilseed legumes due to its short growth period and small genome size. The availability of the high-quality draft genome of adzuki bean allowed us to characterize the RNAi machinery components. In this study, with a comprehensive analysis, a set of DCL, AGO, and RDR genes was characterized in adzuki bean, which supplied basic genomic information for these gene families and provided insights into the probable physiological function of these genes in response to biotic and drought stresses.

## Materials and methods

### Identification of putative *Vigna angularis* DCL, AGO, and RDR genes

The methods for identifying the putative adzuki bean DCL, AGO and RDR genes were as described previously with minor modifications [28]. Generally, the hidden Markov model (HMM) profiles of the DCL, AGO and RDR families were extracted from Pfam (http://www.sanger.ac.uk/). The published Arabidopsis DCL, AGO, and RDR protein sequences were downloaded from the Uniprot database and aligned for conserved domain identification. An HMM profile was generated using the HMMER 2.1.1 software package (http://hmmer.janelia.org/) and was then used to search the adzuki bean database. A nonredundant set of putative sRNA biogenesis proteins identified was further confirmed for the presence of domains specific to each family using SMART and BLASTp searches. The identified genes were designated based on their phylogenetic relationship with their orthologues in chickpea, pigeonpea and soybean. The physio-chemical properties of the identified proteins were calculated using the ExPASy Compute pI/Mw tool.

### Phylogenetic relationship analyses and structure identification

The DCL, AGO and RDR proteins of soybean, chickpea and pigeonpea were downloaded and aligned with Clustal W. A phylogenetic tree was constructed with the neighbor-joining method implemented in MEGA5 [29] with 1,000 bootstrap replicates. The functional protein domains were investigated by SMART and Pfam predictions with the protein sequences as inputs.

### Genes Exon‒Intron structure, potential promoter prediction and miRNA analysis

The exon**‒**intron organization of the adzuki bean DCL, AGO and RDR genes was determined with the online GSDS1.0 program based on the sequence comparison of the full-length coding sequences with their corresponding genomic sequences. The 1.5 kb sequences upstream of the translation initiation codon of all DCLs, AGOs, and RDRs of adzuki bean were extracted and searched for the presence of *cis*-regulatory elements using the PlantCARE database [30]. Only the *cis* elements with a matrix score greater than four were considered. For the identification of miRNAs targeting DCLs, AGOs, and RDRs in adzuki beans, all the miRNAs sequenced in adzuki beans were scanned against the transcript sequences of these genes using psRNATarget [31] and psRobot [32] with default parameters.

### Plant materials and stress treatment

To explore the responses of the DCL, AGO and RDR genes to drought stress, commercial varieties with drought resistance (jinxiaodou 5, supplied by Shanxi Academy of Agricultural Sciences) and drought susceptibility (nongdadai 1, purchased from Beijing Taimin Tongfeng Agricultural Technology Co. Ltd) were used for gene expression analyses. Drought treatments started when the plants reached the first leaf stage by withholding watering, and the control plants were irrigated regularly. The leaves were then collected 15d post drought treatment based on the leaf wilting symptoms. For biotic stress treatment of adzuki bean, all the plants were grown and multiplied under greenhouse conditions and inoculated at the 2–3 leaf stage. *Podosphaera xanthii-*infected powdery mildew of adzuki bean was used to inoculate susceptible (jinxiaodou 5) and slightly resistant (jingnong 26, bred by Beijing University of Agriculture) cultivars by transferring conidia from infected plant leaves to the cultivars to give a density of approximately 5–10 spores/cm^2^ (each plant was inoculated once), and the leaves were then collected 12d post inoculation with visible symptoms. Bean common mosaic virus (BCMV) was propagated in adzuki bean plants and homogenates from BCMV-infected leaves were used to rub the first true leaf of each adzuki bean plant (cultivar jingnong 25, bred by Beijing University of Agriculture) with an equal volume of BCMV-containing homogenate or 0.1 M phosphate buffer (pH 7.0) as a control. The infection of BCMV was confirmed by RT-PCR. All the plants were planted in the greenhouse at Beijing University of Agriculture under the conditions of 14 h daytime, 50% to 70% relative humidity and 27 ± 2 °C.

### Express analyses of DCL, AGO and RDR under different stresses

Total RNA from adzuki bean leaf tissues was isolated with TRIzol reagent (Invitrogen, USA) following the manufacturer′s instructions. The quality and quantity of these total RNA samples were assessed using an Agilent 2100 Bioanalyzer (Agilent Technologies, CA, USA) and a NanoDrop 8000 spectrophotometer (Thermo Scientific, USA). cDNA was synthesized using Invitrogen SuperScript.

RT III First Strand Synthesis Kit according to the manufacturer′s specifications. The gene-specific primers for qRT-PCR were designed using the PrimerQuest tool with default parameters (Supplementary Table 4). qRT-PCRs were conducted with SYBR green mastermix in 96-well plates with three biological replicates using ELF1B as the endogenous control [33]. The PCR conditions used were as follows: 10 min at 95 °C, and 40 cycles of 15 s at 95 °C, and 1 min at 60 °C. The fold-change of the relative transcriptional level in each gene was calculated using the 2^−ΔΔCt^ method. All the data shown are the average means of three independent experiments ± SDs. Data analysis was performed using SPSS software, and significant differences were determined by Student’s t test at significance levels of *P* < 0.001 (***), 0.001 < *P* < 0.01 (**) and 0.01 < *P* < 0.05 (*).

## Results

### In silico identification and phylogenetic analyses of DCL, AGO and RDR proteins

A HMM and BLASTp analyses for the identification of AGO, DCL and RDR proteins were conducted against the adzuki bean genome using the corresponding Arabidopsis protein sequences as queries. The gained candidate sequences were further analysed for conserved domain analyses. Finally, a total of 7 genes encoding DCL (VaDCL), 11 encoding AGO (VaAGO) and 6 encoding RDR (VaRDR) proteins were identified in the adzuki bean genome database (Table 1).

**Table 1:** Sequence characterization of Dicer-like, Argonaute and RNA dependent RNA polymerase genes identified in adzuki bean

Serial no	Gene	Genome coordinates	ORF Length	Protein			Introns
				Length (aa)	Mol. Wt. (Da)	PI	
**DICER-like**							
1	VaDCL1	scaffold635:359,217:372,167	5874	1957	219,311.2	6.76	20
2	VaDCL2a	Chr3:17,402,852:17,413,213	4335	1444	163,687.3	7.23	23
3	VaDCL2b	Chr9:21,152,788:21,160,150	2262	753	85,811.6	6.43	10
4	VaDCL2c	Chr9:23,164,541:23,180,126	4155	1384	157,192.2	7.35	22
5	VaDCL2d	Chr9:23,194,981:23,205,383	3123	1040	118,192.8	6.83	15
6	VaDCL3	Chr4:32,320,440:32,332,783	3891	1296	144,853.2	6.81	17
7	VaDCL4	Chr1:33,082,926:33,098,869	4089	1362	153,586.7	6.58	19
**ARGONAUTES**							
1	VaAGO1	Chr10:4,678,432:4,683,786	3195	1064	117,653.8	9.61	21
2	VaAGO3a	Chr1:18,556,195:18,559,446	3015	1004	112,723.5	9.19	3
3	VaAGO3b	Chr9:23,653,089:23,656,520	2625	874	99,182.9	8.92	2
4	VaAGO4a	Chr7:3,626,555:3,633,798	2892	963	107,595.7	8.81	23
5	VaAGO4b	Chr10:7,424,989:7,431,762	2790	929	103,779.2	8.62	22
6	VaAGO6	Chr8:5,397,780:5,408,800	2232	743	83,015.5	8.05	19
7	VaAGO7a	Chr6:14,871,442:14,875,963	2970	989	113,078.9	9.46	3
8	VaAGO7b	Chr6:14,904,177:14,907,071	2730	909	103,408.6	9.32	2
9	VaAGO10a	Chr1:34,889,624:34,897,482	2766	921	104,373.7	9.14	23
10	VaAGO10b	Chr2:30,116,181:30,123,164	2907	968	108,729.5	9.50	21
11	VaAGO10c	Chr4:4,313,319:4,319,788	2739	912	103,698.1	9.35	22
**RNA-DEPENDENT RNA POLYMERASES**							
1	VaRDR1a	Chr6:18,814,093:18,819,600	3396	1131	128,889.9	7.75	4
2	VaRDR1b	Chr6:18,829,832:18,836,641	3351	1116	127,820.1	8.70	4
3	VaRDR1c	Chr6:18,860,275:18,865,547	3399	1132	129,917.7	8.71	4
4	VaRDR2	Chr1:32,133,579:32,137,755	3360	1119	127,573.9	6.85	4
5	VaRDR3	Chr10:654,766:666,294	2925	974	111,060.8	6.90	18
6	VaRDR6	Chr4:31,005,786:31,009,780	3615	1204	137,237.4	7.85	2

**Fig. 1:**
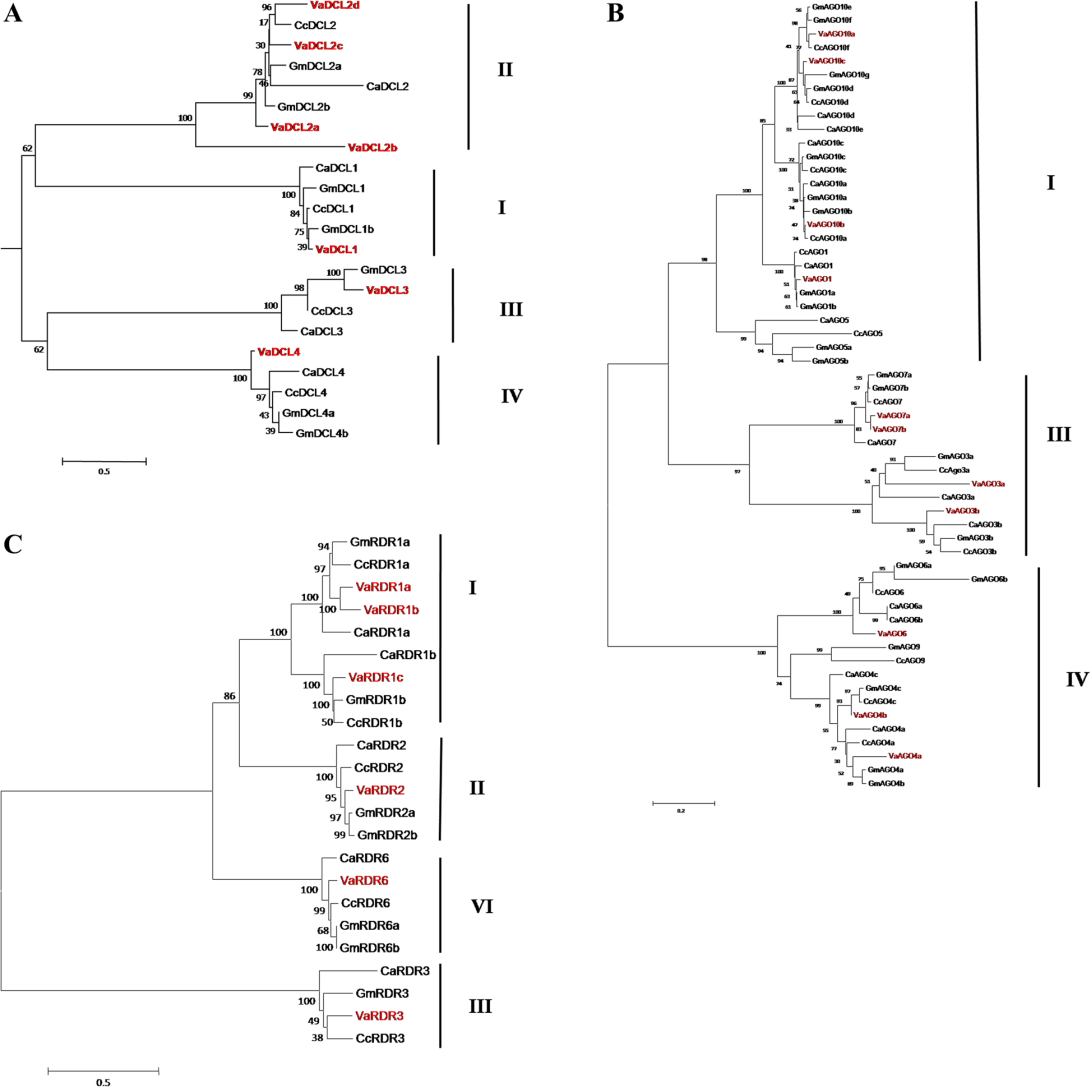
Phylogenetic analysis of DCL, AGO, and RDR proteins in adzuki bean. The tree was constructed by Neighbor-joining method using orthologs from soybean, chickpea and pigeonpea for (**A**) DCL with four; (**B**) AGO with three and (**C**) RDR with four clades. Ca, Cc, Gm and Va represent chickpea, pigeonpea, soybean, and adzuki bean, respectively

In adzuki bean, the DCLs nearly doubled compared with Arabidopsis, pigeonpea and chickpea, similar to soybean. Varied numbers of DCLs are very common and have been reported in other crops [26–28]. Based on the phylogenetic analysis, DCLs were grouped into four clades designated DCL I, DCL II, DCL III, and DCL IV (Fig. 1A), as evidenced for other reported DCLs. Each clade comprised one member except for clade II with four DCLs. In combination with the phylogenetic relationships and sequence homologies with soybean, pigeonpea and chickpea, the 7 VaDCLs were named VaDCL1, VaDCL2a, VaDCL2b, VaDCL2c, VaDCL2d, VaDCL3 and VaDCL4 in accordance with their genomic localization (from a to d). The length of the identified DCLs varied from 753 to 1957 amino acid polypeptides (Table 1), the shortest of which was in clade II, in accordance with a previous report on other legumes.

As inferred from the phylogenetic tree and the protein sequence homologies, the VaAGO family consisted of 1 AGO1 (VaAGO1), 2 AGO3s (VaAGO3a and VaAGO3b), 2 AGO4s (VaAGO4a and VaAGO4b), 1 AGO6 (VaAGO6), 2 AGO7s (VaAGO7a and VaAGO7b) and 3 AGO10s (VaAGO10a, VaAGO10b and VaAGO10c) and they clustered into three clades, AGO I, AGO II, and AGO IV (Fig. 1B). AGO1 and AGO10 were grouped in clade I, AGO3 and AGO7 in clade III, and AGO4 and AGO6 in clade IV respectively. Members of AGO2, AGO5, AGO8, and AGO9 were not identified in adzuki beans. Similar to other legume crops, two AGO3 homologues (AGO3a and AGO3b) were detected in adzuki beans. In chickpea and pigeonpea, AGO3 clustered with Arabidopsis AGO2 and AGO3 and they may functionally compensate for AGO2. In Medicago, soybean, chickpea and pigeonpea, AGO8 was absent, indicating the similar evolution of sRNA biogenesis genes in these legume plants. Compared with other reported legumes, the adzuki bean lacked AGO5, indicating the specific characterization of VaAGOs and their potential function. The length of the candidate AGOs varied from 743 aa to 1,064 aa similar to the AGOs of other legumes (Table 1).

As demonstrated in DCLs and AGOs, RDRs in adzuki beans were named in accordance with the homologues with the closest phylogenetic relationship and highest protein sequence homology. Finally, the adzuki bean genome comprised 3 RDR1 genes (VaRDR1a, VaRDR1b and VaRDR1c), 1 RDR2 gene (VaRDR2), 1 RDR3 gene (VaRDR3) and 1 RDR6 gene (VaRDR6) and clustered into four groups (Fig. 1C).

### Structural organization and conservation patterns

**Fig. 2:**
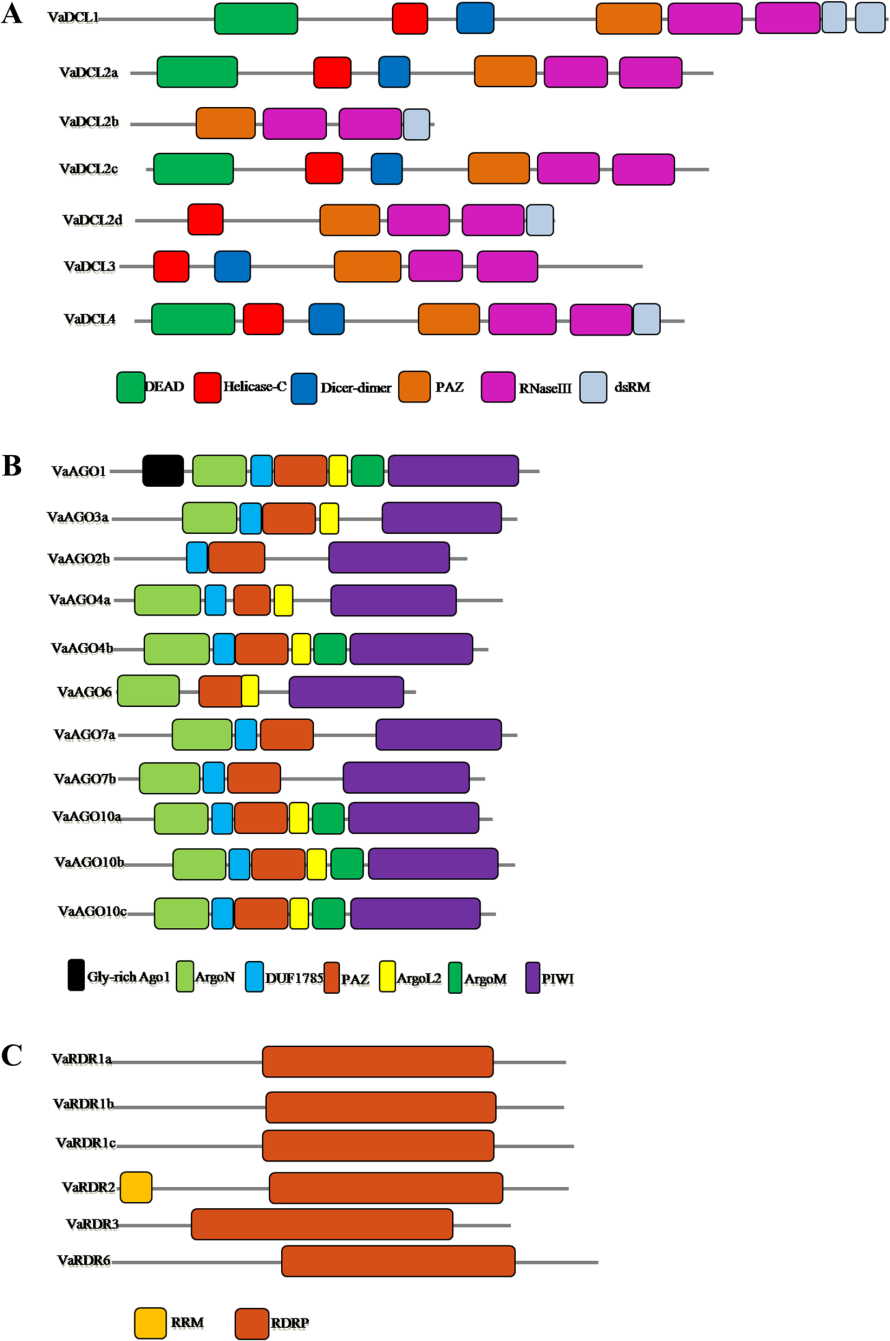
Domain compositions of adzuki bean DCL (**A**), AGO (**B**), and RDR (**C**) protein sequences. Domains are indicated as boxes in various colors. The diagrams were drawn to scale

The physico-chemical properties and conserved domain of adzuki bean DCL, AGO, and RDR proteins were mostly similar to the corresponding proteins in other legume plants with exceptions. Pfam and SMART analyses revealed that VaDCL1 and VaDCL4 contained all the typical domains of the Dicer family (class 3 RNase III family) [34] (Fig. 2A), with the exception of two dsRM domains in VaDCL1, in accordance with the DCL1 in pigeonpea. In the case of chickpea and groundnut, all DCLs except clade II contained two copies of the dsRM domain. The VaDCL2a and VaDCL2c had similar structures that lacked the dsRM domain (Fig. 2A). This was different from Arabidopsis and soybean, in which all clade II DCLs had a single copy of the dsRM domain. In VaDCL2b, only the domains of PAZ, two tandem ribonuclease III and dsRM were present, while VaDCL2d lacked the DEAD and helicase-C. In VaDCL3 the DEAD and dsRM were absent (Fig. 2A). The lack of DEAD and dsRM was also observed in *Brassica napus* DCL3.

The domain composition of VaAGOs was similar to that of other plant AGOs. In addition to the DUF1785, PAZ, ArgoN, and PIWI domains in AGO, additional conserved motifs, including ArgoL and Argomid (mid domain of Argonaute), were also identified in some VaAGOs (Fig. 2B). An additional Gly-rich_AGO1 domain which was reported in AGO1 of Arabidopsis, chickpea, groundnut, pigeonpea and soybean, was also observed in VaAGO1 [27, 35].

In AGOs, the conserved metal-chelating Asp-Asp-His (DDH) motif in the PIWI domain is involved in endonucleic activity. Along with DDH (D760, D845, H986), another conserved residue H798 of AtAGO1 is reported to affect AGO slicer activity. However, studies on many plants, including Arabidopsis, rice, maize, chickpea and soybean, revealed that DDH/H is not a fundamental motif and could be replaced by patterns such as DDD/H, DDH/P, and DDH/S. Multiple sequence alignment of VaAGOs with Arabidopsis AGOs confirmed the presence of a conserved DDH/H motif in the seven VaAGOs, and other motifs such as DDD/H, DDD/N, DDH/P, DDH/S, and DDH/A were also reported in chickpea, groundnut and pigeonpea.

A conserved RdRP domain has been identified in all RDR genes. However, in many plant species including Arabidopsis, soybean, chickpea groundnut, oilseed rape and pigeonpea, an extra conserved RNA recognition motif (RRM) exists in clade I and clade II RDRs. In adzuki bean, this RRM domain was observed only in VaRDR2 (Fig. 2C). The length of the VaRDR ORFs varied from 2,925 bp (VaRDR3) to 3,615 bp (VaRDR6), with coding potentials of 974 and 1,204 amino acids, respectively (Table 1).

### Exon–Intron structure analysis, promoter prediction and miRNA identification

Knowledge of the exon–intron structure of different DCL, AGO, and RDR genes may indicate comprehension of their possible structural evolution process. For VaDCLs, the intron number varied from 10 to 23, and this difference existed in group II. For VaRDRs, most had 2 or 4 introns, while VaRDR3 had 18 introns. In VaAGOs, intron number was relatively conserved among members of each clade but differed greatly between clade III and other clades, similar to that of oilseed plants. A number of *cis*-acting elements were identified in all the members of DCL, AGO, and RDR in the promoter prediction analysis. Light responsive elements, *cis*-acting regulatory elements involved in the hormones or defence and stress responsiveness were the most frequent. *Cis*-acting elements related to circadian control, low-temperature responsiveness, anaerobic induction, zein metabolism regulation and drought inducibility were also observed (Supplemental Tables 1–3). The identification of these *cis*-acting elements indicated the possible roles of DCLs, AGOs and RDRs in the regulation of photoperiodic control of flowering, hormone (salicylic acid, abscisic acid and methyl jasmonate) signalling pathway and stress responses.

All the VaDCLs were recognized as miRNA targets except for VaDCL3 (Figure S1). VaDCL1 was targeted by miRNA162a-3p, VaDCL2a by miR1515a and a novel miRNA miR332, VaDCL2b by miR1515a, VaDCL2c by miR1515a and miR2876-5p and VaDCL4 by miR421, a novel miRNA identified in adzuki bean by deep sequencing. DCL1 targeted by miRNA162 was also reported in the legume species chickpea, pigeonpea and groundnut. MiR1515 targeting DCL2 was also very common in other beans, including soybean, chickpea, pigeonpea and groundnut. Two RDRs in adzuki beans were targeted by miRNAs. VaRDR1b was recognized by miR7810 and RDR2 by miR6029/miR4993. VaAGO7a and VaAGO7b were targeted by miR1850.1, and AGO4b was targeted by miR4993.

### Gene expression analysis in response to drought and biotic stresses

**Fig. 3:**
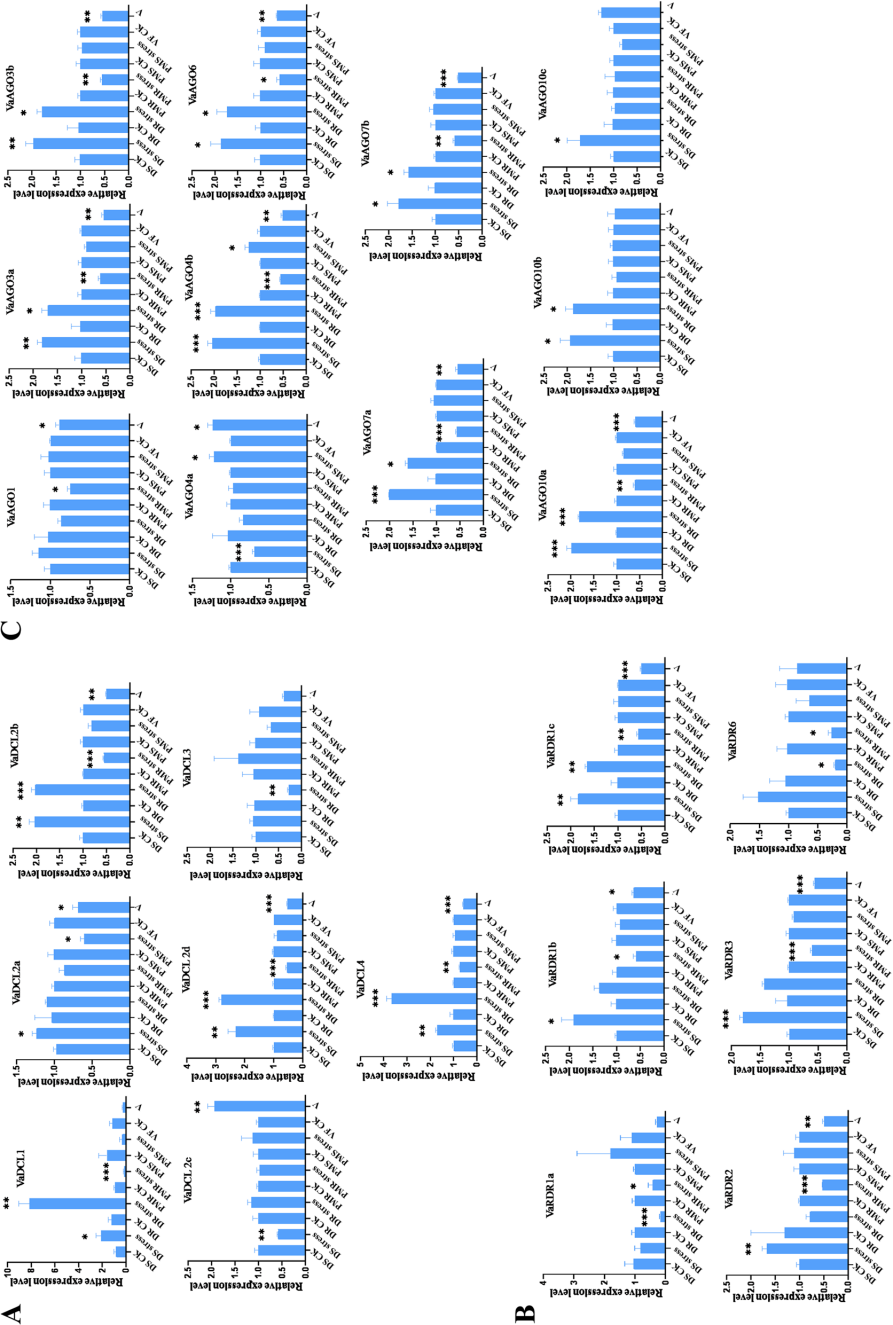
Responses of DCL (**A**), RDR (**B**) and AGO (**C**) in adzuki bean to different stresses. DS: drought-susceptible, DR: drought resistant; PMR: powdery mildew slight resistant, PMS: powdery mildew susceptible;VF: virus free; V: virus-infected; CK:control. Error bars show the standard error between three replicates performed. Asterisks indicate significant differences with Student’s t test. *P* < 0.001 (***), 0.001 < *P* < 0.01 (**) and 0.01 < *P* < 0.05 (*)

To explore the potential role of the DCL, AGO, and RDR genes in the posttranscriptional regulation of gene expression in response to drought and biotic stresses, the expression patterns of all these genes under drought, *Podosphaera xanthii* and BCMV infection were analysed by qRT-PCR. VaDCL1/2b/2d/4 were significantly upregulated under drought stress in both resistant (DR) and susceptible (DS) genotypes, but downregulated in the powdery mildew slightly resistant (PMR) genotype challenged with *Podosphaera xanthii* (Fig. 3A). The downregulation of VaDCL2c was observed in the DS genotype and VaDCL3 in the DR genotype (Fig. 3A). VaDCL2a/2b/2d/4 were downregulated in BCMV-infected plants, but VaDCL2c was upregulated (Fig. 3A). Under drought stress, the expression levels of VaRDR1a and VaRDR6 were depressed in the DR genotype (Fig. 3B). However, VaRDR1b/2/3 in DS genotype and VaRDR1c in both genotypes were all upregulated (Fig. 3B). All the VaRDRs in the *Podosphaera xanthii*-infected PMR genotype and VaRDR1b/1c/2/3 in BCMV-infected plants were downregulated (Fig. 3B). All VaAGOs were upregulated under drought stress, except for the VaAGO in both genotypes and VaAGO4a and VaAGO10c in the DR genotype, which showed no obvious change (Fig. 3C). In contrast, all VaAGOs were downregulated in the *Podosphaera xanthii* infected PMR genotype and BCMV-infected plants, with the exceptions of VaAGO10b and VaAGO10c, with noticeable changes and upregulation of VaAGO4a and VaAGO4b in the PMS genotype and VaAGO4a under BCMV infection (Fig. 3C). These genes exhibited varying expression patterns under drought and *Podosphaera xanthii* and BCMV infection, indicating the diverse and complex roles of RNA silencing key components in the regulation of adzuki bean to resist drought and biotic stresses.

## Discussion

RNA silencing is a universal molecular mechanism that regulates diverse biological processes through small RNAs produced through the RNA silencing machinery. In this study, the key elements of DCL, AGO and RDR family genes were identified and their relationships with the corresponding genes reported in other legume plants were investigated, which would help us understand the RNA silencing machinery evolution in legumes. Further expression changes in these genes under drought, *Podosphaera xanthii* and BCMV stresses were investigated, indicating the potential roles of these genes in fighting against drought and biotic factors in adzuki bean.

DCL, AGO and RDR genes were identified in the legume plants including soybean [27], chickpea, pigeonpea, chickpea, groundnut [28] and *Medicago truncatula* [36]. For DCLs, there were three in *Medicago truncatula*, four in chickpea, pigeonpea and groundnut, but nearly doubled (seven) in soybean and adzuki bean. Phylogenetic analyses showed that all of them belonged to the four clades, but the number of genes in each clade differed. Clade II VaDCL is composed of four members, which underwent significant expansion in legumes. Similar DCL2 was also reported in *Solanum commersonii*, *Solanum tuberosum*, *Solanum lycopersicum* and *Solanum pennellii* [37, 38]. Tandem and segmental duplications represent the two major forces driving gene duplication [39], and their roles in VaDCL2 duplication need to be further explored. In contrast to the well-documented DCL proteins in Arabidopsis, little is known about their action in adzuki bean, especially the diversification of VaDCL2; thus, further study of the biochemical or genetic analysis of DCL genes in adzuki bean is needed. Studies from Arabidopsis, rice, maize, soybean, and chickpea have shown that plants possess four groups of RDRs: RDR1, RDR2, RDR3 and RDR6, which were also identified in adzuki bean. The function of RDRs in some plants has been established, and they appear to be evolutionarily conserved; for example, RDR6 in Arabidopsis and rice is associated with siRNA and tasiRNA in the regulation of endogenous genes [40, 41], and the RDR2 in Arabidopsis and maize is involved in siRNA mediated de novo methylation of direct repeats [42]**.** All these results indicated the conserved gene function of plant RDRs and supported the deduction that the RDR gene family in plants was derived from a common ancestor. It has been proposed that both plants and animals encode multiple AGOs to meet the diversified functions of small RNA silencing [43]. Similar to rice, maize, Arabidopsis, soybean, sorghum and chickpea, adzuki bean also encoded three subfamilies of AGO proteins, indicating that small RNA functions are conserved in higher plants.

Plants utilize RNA silencing machinery to facilitate pathogen-associated molecular pattern-triggered immunity and effector-triggered immunity to defend against pathogen attack or to facilitate defense against insect herbivores, or other abiotic stresses, including drought, cold and mineral nutrient deficiency, dehydration, and even mechanical stimuli [44]. DCLs and AGOs initiated and maintained RNA silencing, while RDRs amplified the signal to generate secondary small RNAs to provoke a new round of RNA silencing. Drought is one of the main environmental factors that threatens agricultural production. The responses of DCLs, RDRs and AGOs from different plant species to drought have also been widely investigated [45–49]. Upregulation of SsDCL1a/2/3, SsRDR3/6b and SsAGO2b/5a/5c/6b/SsAGO10c [46], ZmDCL2/3b, ZmAGO4a/4d/7, ZmRDR2/4 [48], CaDCL1/2/3, CaRDR2/6, and CaAGO2/10b [49] was observed under drought stress. In our study, four DCLs were significantly upregulated and two (VaDCL2c in DS and VaDCL3 in DR) were downregulated, similar to the expression pattern in peach [47]. Several AGOs were induced while VaAGO4a was depressed in the DS genotype (Fig. 3C). SlAGO4a-downexpression transgenic tomato appeared to have enhanced drought tolerance with strong photosynthetic capability, more membrane integrity and stability as well as better cell viability and more efficient antioxidant defence machinery [50]. Thus AGO4a expression may be a negatively correlated with tolerance to drought.

The role of RNA silencing in plant defence against viruses has been well documented, and increasing evidence shows that RNA silencing is also involved in plant-bacteria/fungi interactions, which has been reported in plants fighting against *Pseudomonas syringae* [51], *Colletotrichum lindemuthianum* [52], *Verticillium dahliae* [53], *Sclerotinia sclerotiorum* [54] and *Botryosphaeria dothidea* [55]. MhAGO1 was demonstrated to be a negative regulator of *B. dothidea* resistance, and delayed symptom development was observed in MhAGO1-silenced leaves [55]. Similar reduced symptoms were also observed in the Arabidopsis ago1 mutant but were enhanced in the ago7, dcl4, rdr2 and rdr6 mutants [53]. In adzuki bean, most of the RNA silencing downregulation of VaDCL1/2b/2d/4, VaRDR1b/1c/2/3 and VaAGO1/3a/3b/4b/6/7a/7b/10a was observed in the PMR genotype. The expression change patterns of VaDCLs, VaAGOs and VaRDRs in BCMV infected plants were nearly the same as those demonstrated under *Podosphaera xanthii* infection (Fig. 3). The different responses of VaDCLs, VaRDRs and VaAGOs to drought and biotic stresses may be related to the unique roles of these genes in contributing to drought and biotic stress resistance.

MiRNAs are involved in plant responses to various stresses through regulation of target gene expression [13, 14]. The biogenesis of miRNAs is dependent on the regulatory proteins, such as AGO, DCL and RDR; in turn, these regulatory proteins are also regulated by certain miRNAs [56]. This feedback regulation has been reported in plant responses to both biotic and abiotic stresses, including repression of AGO1 by miR168 during virus infection in *Nicotiana benthamiana* [57], upregulation of miR168 for AGO1 degradation under drought in Arabidopsis [58], degradation of DCL1, AGO1 and AGO2 by miR162a, miR168a and miR403 respectively in *Phaseolus vulgaris* under different stresses [59], downregulation of AGO1, AGO2, DCL1 and DCL2 by miR168a, miR403a, miR162b and miR1515a respectively in soybean infected by soybean mosaic virus [60]. In this study, VaAGO, VaDCL and VaRDR were also predicted to be targeted by some miRNAs, and the feedback regulation of these proteins by miRNAs in adzuki bean under drought and biotic stresses should also be considered.

The identification of these putative RNA silencing components would provide insight into small RNA pathways in adzuki bean. However, the exact function and contribution of individual components of the RNA silencing machinery in regulating biotic or drought stresses needs to be further explored, which would assist in the breeding of stress resistance in adzuki bean.

## Supplementary Information

**Additional file 1:** **Figure S1.** MiRNA targets identified by psRobot. **Table S1.** Promoter analysis of VaDCLs. **Table S2.** Promoter analysis of VaAGOs. **Table S3.** Promoter analysis of VaRDRs. **Table S4.** Primers used in the QRT-PCR analysis.

## Data Availability

This Whole Genome Shotgun project has been deposited at GenBank under the accession no. PRJNA261643. Materials described in the manuscript, including all relevant raw data, are freely available to any scientist wishing to use them for non-commercial purposes upon request via e-mail with the corresponding author.

## References

[1] Yang K, Tian Z, Chen C, Luo L, Zhao B, Wang Z, Yu L, Li Y, Sun Y, Li W (2015). Genome sequencing of adzuki bean (Vigna angularis) provides insight into high starch and low fat accumulation and domestication. Proc Natl Acad Sci USA.

[2] Wang Y, Yao X, Shen H, Zhao R, Li Z, Shen X, Wang F, Chen K, Zhou Y, Li B, Zheng X, Lu S (2022). Nutritional composition, efficacy, and processing of Vigna angularis (Adzuki Bean) for the human diet: An overview. Molecules.

[3] Singh N, Kharwal N, Bhardwaj N, Singh S. (2023). Chapter 20-Adzuki bean [Vigna angularis (willd.) Ohwi & Ohashi] in Neglected and Underutilized Crops, eds. Farooq, M., Siddique, K.H.M., Academic Press, 539–556.

[4] Shahrajabian MH, Sun WL, Cheng Q. (2022). Chapter 20-A survey of biological nitrogen fixation in adzuki beans, soybeans, and mung beans, three legumes in traditional Chinese medicine in Functional Foods and Nutraceuticals in Metabolic and Non-Communicable Diseases, eds. Singh, R.B., Watanabe, S., Isaza, A.A., Academic Press, 301–316

[5] Zhang J, Xie X, Zhang L, Hong Y, Zhang G, Lyu F (2022). Optimization of microwave pre-cooked conditions for gelatinization of adzuki bean. Foods.

[6] Amarowicz R, Estrella I, Hernández T, Troszyńska A (2008). Antioxidant activity of extract of adzuki bean and its fractions. J Food Lipids.

[7] Yao Y, Cheng X, Wang S, Wang L, Ren G (2012). Influence of altitudinal variation on the antioxidant and antidiabetic potential of adzuki bean (Vigna angularis). Int J Food Sci Nutr.

[8] Li Y, Zhang Q, Zhang J, Wu L, Qi Y, Zhou JM (2010). Identification of microRNAs involved in pathogen-associated molecular pattern-triggered plant innate immunity. Plant Physiol.

[9] Wang L, Wang J, Cheng X, Al-Khayri J, Jain S, Johnson D (2019). Adzuki Bean (Vigna angularis (Willd.) Ohwi & Ohashi) Breeding. Advances in Plant Breeding Strategies: Legumes.

[10] Qin JC, Yin Z, Shen DY, Chen HT, Chen X, Cui XY, Chen XH (2021). Development of a recombinase polymerase amplification combined with lateral flow dipstick assay for rapid and sensitive detection of bean common mosaic virus. Phytopathol Res.

[11] Li YQ, Liu ZP, Yang K, Li YS, Zhao B, Fan ZF, Wan P (2014). First Report of Bean common mosaic virus Infecting Azuki Bean (Vigna angularis) in China. Plant Dis.

[12] Tayade R, Rana V, Shafiqul M, Nabi RBS, Raturi G, Dhar H, Thakral V, Kim Y (2022). Genome-wide identification of aquaporin genes in adzuki bean (Vigna angularis) and expression analysis under drought stress. Int J Mol Sci.

[13] Chen XM (2009). Small RNAs and their roles in plant development. Annu Rev Cell Dev Biol.

[14] Ruiz-Ferrer V, Voinnet O (2009). Roles of plant small RNAs in biotic stress responses. Annu Rev Plant Biol.

[15] Meins F, Si-Ammour A, Blevins T (2005). RNA silencing systems and their relevance to plant development. Annu Rev Cell Dev Biol.

[16] Hannon GJ (2002). RNA interference. Nature.

[17] Moazed D (2009). Small RNAs in transcriptional gene silencing and genome defense. Nature.

[18] Hutvagner G, Simard MJ (2008). Argonaute proteins: key players in RNA silencing. Nat Rev Mol Cell Biol.

[19] Sijen T, Fleenor J, Simmer F, Thijssen KL, Parrish S, Timmons L, Plasterk RHA, Fire A (2001). On the role of RNA amplification in dsRNA-triggered gene silencing. Cell.

[20] Bologna NG, Voinnet O (2014). The diversity, biogenesis, and activities of endogenous silencing small RNAs in Arabidopsis. Annu Rev Plant Biol.

[21] Mallory A, Vaucheret H (2010). Form, function, and regulation of ARGONAUTE proteins. Plant Cell.

[22] Zheng XW, Zhu JH, Kapoor A, Zhu JK (2007). Role of Arabidopsis AGO6 in siRNA accumulation, DNA methylation and transcriptional gene silencing. EMBO J.

[23] Montgomery TA, Howell MD, Cuperus JT, Li DW, Hansen JE, Alexander AL, Chapman EJ, Fahlgren N, Allen E, Carrington JC (2008). Specificity of ARGONAUTE7-miR390 interaction and dual functionality in TAS3 trans-acting siRNA formation. Cell.

[24] Katiyar-Agarwal S, Gao S, Vivian-Smith A, Jin H (2007). A novel class of bacteria-induced small RNAs in Arabidopsis. Genes Dev.

[25] Yoshikawa M, Peragine A, Park MY, Poethig RS (2005). A pathway for the biogenesis of trans-acting siRNAs in Arabidopsis. Genes Dev.

[26] Cao JY, Xu YP, Li W, Li SS, Rahman H, Cai XZ (2016). Genome-wide identification of dicer-like, argonaute, and RNA-dependent RNA polymerase gene families in brassica species and functional analyses of their arabidopsis homologs in resistance to sclerotinia sclerotiorum. Front Plant Sci.

[27] Liu X, Lu T, Dou YC, Yu B, Zhang C (2014). Identification of RNA silencing components in soybean and sorghum. BMC Bioinformatics.

[28] Garg V, Agarwa G, Pazhamala LT, Nayak SN, Kudapa H, Khan AW, Doddamani D, Sharma M, KaviKishor PB, Varshney RK (2017). Genome-wide identification, characterization, and expression analysis of small RNA biogenesis purveyorsreveal their role in regulation of biotic stress responses in three legume crops front. Plant Sci.

[29] Tamura K, Peterson D, Peterson N, Stecher G, Nei M, Kumar S (2011). MEGA5: molecular evolutionary genetics analysis using maximum likelihood, evolutionary distance, and maximum parsimony methods. Mol Biol Evol.

[30] Lescot M, Dehais P, Thijs G, Marchal K, Moreau Y, Van de Peer Y (2002). PlantCARE, a database of plant cis-acting regulatory elements and a portal to tools for in silico analysis of promoter sequences. Nucleic Acids Res.

[31] Dai X, Zhao PX (2011). psRNATarget: a plant small RNA target analysis server. Nucleic Acids Res.

[32] Wu HJ, Ma YK, Chen T, Wang M, Wang XJ (2012). PsRobot: a web-based plant small RNA meta-analysis toolbox. Nucleic Acids Res.

[33] Wang J, Yang K, Zhao B, Li YS, Wan P (2020). Identification and validation of reference genes in the adzuki bean (Vigna angularis) under iron deficiency using quantitative real-time PCR. Plant Mol Biol Rep.

[34] MacRae IJ, Doudna JA (2007). Ribonuclease revisited: structural insights into ribonuclease III family enzymes. Curr Opin Struc Biol.

[35] Xie Z, Johansen LK, Gustafson AM, Kasschau KD, Lellis AD, Zilberman D (2004). Genetic and functional diversification of small RNA pathways in plants. PLoS Biol.

[36] Capitão C, Paiva LAP, Santos DM, Fevereiro P (2011). In Medicago truncatula, water deficit modulates the transcript accumulation of components of small RNA pathways. BMC Plant Biol.

[37] Bai M, Yang GS, Chen WT, Mao ZC, Kang HX, Chen GH, Yang YH, Xie BY (2012). Genome-wide identification of Dicer-like, Argonaute and RNA-dependent RNA polymerase gene families and their expression analyses in response to viral infection and abiotic stresses in Solanum lycopersicum. Gene.

[38] Esposito S, Aversano R, D’Amelia V, Villano C, Alioto D, Mirouze M, Carputo D (2018). Dicer-like and RNA-dependent RNA polymerase gene family identification and annotation in the cultivated Solanum tuberosum and its wild relative S. commersonii. Planta.

[39] Kong HZ, Landherr LL, Frohlich MW, Leebens-Mack J, Ma H, DePamphilis CW (2007). Patterns of gene duplication in the plant SKP1 gene family in angiosperms: evidence for multiple mechanisms of rapid gene birth. Plant J.

[40] Luo ZH, Chen ZX (2007). Improperly terminated, unpolyadenylated mRNA of sense transgenes is targeted by RDR6-mediated RNA silencing in arabidopsis. Plant Cell.

[41] Nagasaki H, Itoh J, Hayashi K, Hibara K, Satoh-Nagasawa N, Nosaka M, Mukouhata M, Ashikari M, Kitano H, Matsuoka M, Nagato Y, Sato Y (2007). The small interfering RNA production pathway is required for shoot meristem initiation in rice. Proc Natl Acad Sci USA.

[42] Alleman M, Sidorenko L, McGinnis K, Seshadri V, Dorweiler JE, White J, Sikkink K, Chandler VL (2006). An RNA-dependent RNA polymerase is required for paramutation in maize. Nature.

[43] Bartel DP (2004). MicroRNAs: genomics, biogenesis, mechanism, and function. Cell.

[44] Huang J, Yang ML, Zhang XM (2016). The function of small RNAs in plant biotic stress response. J Integr Plant Biol.

[45] Balassa G, Balassa K, Janda T, Rudnóy S (2022). Expression pattern of RNA interference genes during drought stress and MDMV infection in maize. J Plant Growth Regul.

[46] Cui DL, Meng JY, Ren XY, Yue JJ, Fu HY, Huang MT, Zhang QQ, Gao SJ (2020). Genome-wide identification and characterization of DCL, AGO and RDR gene families in Saccharum spontaneum. Sci Rep.

[47] Belal MA, Ezzat M, Zhang YQ, Xu Z, Cao YP, Han YP (2022). Integrative analysis of the DICER-like (DCL) genes from peach (Prunus persica): A Critical role in response to drought stress. Front Ecol Evol.

[48] Qian YX, Cheng Y, Cheng X, Jiang HY, Zhu SW, Cheng BJ (2011). Identification and characterization of Dicer-like, Argonaute and RNA-dependent RNA polymerase gene families in maize. Plant Cell Rep.

[49] Qin L, Mo N, Muhammad T, Liang Y (2018). Genome-wide analysis of DCL, AGO, and RDR gene families in pepper (Capsicum Annuum L.). Int J Mol Sci.

[50] Huang W, Xian Z, Hu G, Li ZG (2016). SlAGO4A, a core factor of RNA-directed DNA methylation (RdDM) pathway, plays an important role under salt and drought stress in tomato. Mol Breeding.

[51] Agorio A, Vera P (2007). ARGONAUTE4 Is Required for Resistance to Pseudomonas syringae in Arabidopsis. Plant Cell.

[52] Alvarez-Diaz JC, Richard MMS, Thareau V, Teano G, Paysant-Le-Roux C, Rigaill G, Pflieger S, Gratias A, Geffroy V (2022). Genome-wide identification of key components of RNA silencing in two phaseolus vulgaris genotypes of contrasting origin and their expression analyses in response to fungal infection. Genes.

[53] Ellendorff U, Fradin EF, De Jonge R, Thomma BPHJ (2009). RNA silencing is required for Arabidopsis defence against Verticillium wilt disease. J Exp Bot.

[54] Zhao X, Zheng WH, Zhong ZH, Chen XT, Wang AR, Wang ZH (2016). Genome-wide analysis of RNA-interference pathway in Brassica napus, and the expression profile of BnAGOs in response to Sclerotinia sclerotiorum infection. Eur J Plant Pathol.

[55] Yu X, Hou Y, Chen W, Wang S, Wang P, Qu S (2017). Malus hupehensis miR168 targets to ARGONAUTE1 and contributes to the resistance against Botryosphaeria dothidea infection by altering defense responses. Plant Cell Physiol.

[56] Arikit S, Zhai J, Meyers BC (2013). Biogenesis and function of rice small RNAs from non-coding RNA precursors. Curr Opin Plant Biol.

[57] Várallyay É, Válóczi A, Ágyi Á, Burgyán J, Havelda Z (2010). Plant virus-mediated induction of miR168 is associated with repression of ARGONAUTE1 accumulation. EMBO J.

[58] Liu HH, Tian X, Li YJ, Wu CA, Zheng CC (2008). Microarray-based analysis of stress-regulated microRNAs in Arabidopsis thaliana. RNA.

[59] Arenas-Huertero C, Perez B, Rabanal F, Blanco-Melo D, De la Rosa C, Estrada-Navarrete G, Sanchez F, Covarrubias AA, Reyes JL (2009). Conserved and novel miRNAs in the legume Phaseolus vulgaris in response to stress. Plant Mol Biol.

[60] Bao D, Ganbaatar O, Cui X, Yu R, Bao W, Falk BW, Wuriyanghan H (2018). Downregulation of genes coding for core RNAi components and disease resistance proteins via corresponding microRNAs might be correlated with successful SMV infection in soybean. Mol Plant Pathol.

