# Supplementary material for: Genome-wide analysis of key gene families in RNA silencing and their responses to biotic and drought stresses in adzuki bean
Source: BMC Genomics. 2023 Apr 12;24:195. doi: 10.1186/s12864-023-09274-9 (PMC10091639; doi:10.1186/s12864-023-09274-9)
Supplement: Supplementary file 1 — Additional file 1: Figure S1. MiRNA targets identified by psRobot. Table S1. Promoter analysis of VaDCLs. Table S2. Promoter analysis of VaAGOs. Table S3. Promoter analysis of VaRDRs. Table S4. Primers used in the QRT-PCR analysis. [file 12864_2023_9274_MOESM1_ESM.zip › supplemental Figure and table/Figure S1 miRNA targets of adzuki bean DCL, AGO and RdRp identified by psRobot.docx]

**MiRNA targets identified by psRobot**

>miR1515a Score: 2.5 VaDCL2a

Query: 1 TCATTTTGCGTGCAATGATCTG 22

|||||||**|||||||||||::

Sbjct: 989 AGTAAAAAACACGTTACTAGGT 96

>miR1515a Score: 1.5 VaDCL2b

Query: 1 TCATTTTGCGTGCAATGATCTG 22

|||||:||||:|||||||||::

Sbjct: 3074 AGTAAGACGCGCGTTACTAGGT 3053

>miR1515a Score: 2.0 VaDCL2d

Query: 1 TCATTTTGCGTGCAATGATCTG 22

|||||||**|||||||||||||

Sbjct: 2903 AGTAAAAGACACGTTACTAGAC 2882

>miR4993 Score: 2.5 VaRDR2

Query: 1 GAGGAGGTGGCGGTGGAGGAG 21

|||||:||||*:|||||*|||

Sbjct: 258 CTCCTTCACCCTCACCTACTC 238

>miR6029 Score: 2.5 VaRDR2

Query: 1 TGGGGTTGAGTTAGGCTT 18

|||*:|||||||*|||||

Sbjct: 526 ACCGTAACTCAAACCGAA 509

MiRNA targets identified by psRNATarget

query=miR1850.1, target=VaAGO7b, score=4, range=1354-1373, strand=-1

target 5' CCUCC-AUCUCCAAGCUUUAG 3'

:: :: ::::::::.:::::

query 3' GGUGGUUAGAGGUUUGAAAUG 5'

query=miR1850.1, target= VaAGO7a, score=4, range=1594-1613, strand=-1

target 5' CCUCC-AUCUCCAAGCUUUAG 3'

:: :: ::::::::.:::::

query 3' GGUGGUUAGAGGUUUGAAAUG 5'

query=miR7810, target=VaRDR1b, score=4, range=330-350, strand=-1

target 5' AAAACAGAAAAUCCUCCUUUC 3'

: :::::::::::::::.:

query 3' CUCUGUCUUUUAGGAGGAGAA 5'

query=miR162a-3p, target=VaDCL1, score=2, range=3196-3217, strand=1

target 5' CUGGAUGCAGAGGUGUUAUCGA 3'

:::::::::::::: :::::::

query 3' GACCUACGUCUCCA-AAUAGCU 5'

query=novel_mir_332, target=VaDCL2b, score=3.5, range=3369-3389, strand=-1

target 5' AUAAUCAACUCUU-CUAUUUUA 3'

::::.::: :::: ::::::::

query 3' UAUUGGUUCAGAAUGAUAAAAU 5'

>miR2876-5p Score: 0.0 VaDCL2c

Query: 1 AATTGACTGGCAGCATATTTT 21

|||||||||||||||||||||

Sbjct: 3020 TTAACTGACCGTCGTATAAAA 3000

>novel_mir_421 Score: 2.2 VaDCL4

Query: 1 TTCTGGAGCTGCATTATTGTT 21

||||||*|||||||:||:*||

Sbjct: 1572 AAGACCGCGACGTAGTAG-AA 1553

>miR4993 Score: 2.5 VaRDR2

Query: 1 GAGGAGGTGGCGGTGGAGGAG 21

|||||:||||*:|||||*|||

Sbjct: 258 CTCCTTCACCCTCACCTACTC 238

Figure S1 miRNA targets of adzuki bean DCL, AGO and RdRp identified by psRobot
